# Risk Factors for Developing Metachronous Superficial Gastric Epithelial Neoplasms after Endoscopic Submucosal Dissection

**DOI:** 10.3390/jcm13061587

**Published:** 2024-03-10

**Authors:** Tsunehiro Suzuki, Kenichi Goda, Manabu Ishikawa, Shintaro Yamaguchi, Tomonori Yoshinaga, Masayuki Kondo, Mimari Kanazawa, Yasuhito Kunogi, Takanao Tanaka, Akira Kanamori, Keiichiro Abe, Akira Yamamiya, Takeshi Sugaya, Keiichi Tominaga, Hidetsugu Yamagishi, Hironori Masuyama, Atsushi Irisawa

**Affiliations:** 1 Department of Gastroenterology, Dokkyo Medical University School of Medicine, 880 Kitakobayashi Mibu, Tochigi 321-0293, Japan; s-t-hiro@dokkyomed.ac.jp (T.S.); d-ishi@dokkyomed.ac.jp (M.I.); y-shin-y@dokkyomed.ac.jp (S.Y.); tomo-y@dokkyomed.ac.jp (T.Y.); m-kondo@dokkyomed.ac.jp (M.K.); mimari77@dokkyomed.ac.jp (M.K.); ykunogi@dokkyomed.ac.jp (Y.K.); tana1986@dokkyomed.ac.jp (T.T.); k-akira@dokkyomed.ac.jp (A.K.); abe9841@dokkyomed.ac.jp (K.A.); akira-y@dokkyomed.ac.jp (A.Y.); t-sugaya@dokkyomed.ac.jp (T.S.); tominaga@dokkyomed.ac.jp (K.T.); irisawa@dokkyomed.ac.jp (A.I.); 2 Department of Diagnostic Pathology, Dokkyo Medical University School of Medicine, 880 Kitakobayashi Mibu, Tochigi 321-0293, Japan; yamagisi@dokkyomed.ac.jp; 3 Masuyama Gastroenterology Clinic, 83-413 Kajiya Otawara, Tochigi 324-0046, Japan; mgic@titan.ocn.ne.jp

**Keywords:** stomach, early gastric cancer, gastric adenoma, metachronous cancer, cumulative incidence, endoscopic resection, endoscopic submucosal dissection, *Helicobacter pylori*, spontaneous disappearance, intestinal metaplasia

## Abstract

**Background:** Although endoscopic submucosal dissection (ESD) provides a high rate of curative resection, the remaining gastric mucosa after ESD is at risk for metachronous superficial gastric epithelial neoplasms (MSGENs). It leaves room for risk factors for developing MSGENs after ESD. This study aimed to identify clinicopathological risk factors for the occurrence of MSGENs, and to evaluate the association of *Helicobacter pylori* (*H. pylori*) with the MSGENs. **Methods:** We conducted a retrospective cohort study including 369 patients with 382 lesions that underwent ESD for adenoma/early gastric cancer. **Results:** Twenty-seven MSGENs occurred. The subjects were divided into MSGEN and not-MSGEN groups. There was a significantly higher frequency of histological intestinal metaplasia (HIM) and initial neoplasm location in the upper or middle parts (INUM) in the MSGEN group. The HIM and INUM groups had a significantly higher cumulative incidence of MSGENs. We compared 27 patients from the MSGEN group and 27 patients from the not-MSGEN group that were matched to the MSGEN group for variables including HIM and INUM. There was a significantly higher frequency of the spontaneous disappearance of *H. pylori* in the MSGEN group. **Conclusions:** HIM, INUM, and the spontaneous disappearance of *H. pylori* may be clinicopathological risk factors for developing MSGENs after ESD.

## 1. Introduction

Gastric cancer is the sixth most common cancer worldwide [1]. It has a high prevalence in East Asia, with approximately 75% of gastric cancer cases worldwide occurring in East Asia. Gastric cancer is the third-highest main cause of all cancer deaths in Japan, and the widespread use of endoscopy and diagnostic advances have allowed for the early detection of gastric cancer. Endoscopic resection (ER) is a well-known treatment for early gastric cancer. Recently, the rapid widespread adoption of a new ER technique, endoscopic submucosal dissection (ESD), has led to this being performed in between 60% and 70% of all gastric cancer cases [2]. Similar to open surgery, ESD allows en bloc resection regardless of the lesion size if the superficial cancer has a negligible risk for lymph node metastasis. The most important merit of endoscopic treatment, including ESD, is the preservation of the stomach that can offer a significantly better quality of life compared to surgical resection. However, it involves the disadvantage of the remaining gastric mucosa having a high risk of carcinogenesis. Specifically, metachronous gastric cancers following ER have an occurrence rate of 2.5–14% [3,4,5,6].

Recent largescale cohort studies showed a reduced risk of developing metachronous cancer (included in MSGENs) following *Helicobacter pylori* (*H. pylori*) therapy after endoscopic resection of early gastric cancer and adenoma [7,8,9]; therefore, *H. pylori* therapy is recommended for *H. pylori*-positive patients who have undergone ER of the superficial gastric epithelial neoplasms (SGENs). Contrastingly, other studies have reported that the risk of developing metachronous cancer does not decrease significantly after *H. pylori* therapy [5,6,10]. Thus, there is controversy regarding whether *H. pylori* therapy is associated with the risk of developing MSGENs following ER, particularly ESD which has been widely used in recent times. It still leaves room for clinicopathological risk factors for developing MSGENs, including *H. pylori* therapy, especially after ESD.

Studies from Japan demonstrated that early gastric cancer was rarely derived from gastric mucosa with previous *H. pylori* infection-induced atrophic gastritis (e.g., early gastric cancer cases having no history of *H. pylori* therapy and being negative for *H. pylori* tests but having severe gastric gastritis including cases with the spontaneous disappearance of *H. pylori*) at a frequency of 10% or 13.8% in patients with early gastric cancer treated by endoscopy [11,12]. It is noteworthy that the risk of gastric cancer development in cases with the spontaneous disappearance of *H. pylori* with no history of *H. pylori* therapy and severe atrophy (0.53–0.87% per year [13,14,15,16]) was slightly higher than that in post-*H. pylori* therapy cases with severe atrophy (0.31–0.62% per year [13,17,18,19]). There is little known about the associations between the spontaneous disappearance of *H. pylori* and MSGENs following ESD.

This retrospective cohort study aimed to identify the clinicopathological risk factors, including the spontaneous disappearance of *H. pylori*, for developing MSGENs in patients who underwent ESD for SGENs including MSGENs after previous ESD.

## 2. Materials and Methods

### 2.1. Patients

We identified 519 patients (531 lesions) who underwent ESD for SGENs at DokkyoMedical University Hospital from January 2014 to December 2018. We performed abdominal/chest CT for all of the patients. The criteria for ESD were superficial gastric neoplasms involving gastric adenoma suspected of being cancerous and differentiated type adenocarcinomas in situ or with slight invasion to the submucosa with no evidence of lymph node metastasis on preoperative CT (abdomen and chest). 

**Figure 1:**
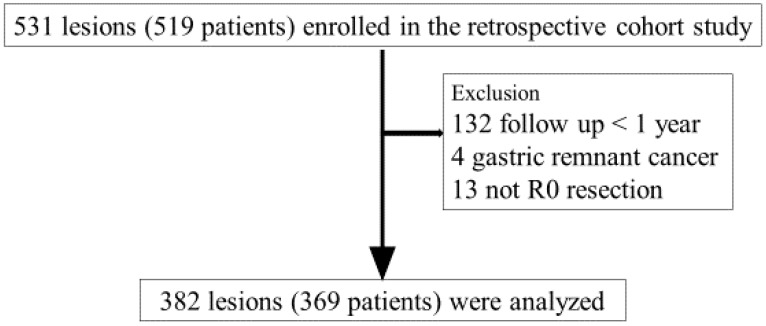
Flowchart of study patients.

We excluded 132 patients who did not undergo endoscopic follow-up observation within <1 year after ESD, 4 patients with gastric remnant cancer, and 13 patients who did not undergo R0 resection; finally, we included 369 patients (382 lesions) (Figure 1). This study did not include patients with gastrointestinal polyposis syndromes (e.g., gastric adenocarcinoma and proximal polyposis of the stomach, familial adenomatous polyposis, and Juvenile polyposis syndrome).

The criteria for additional surgery after ESD were non-R0 resection corresponding to cancerous lesions with massive invasion to the submucosa deeper than 500 μm, being positive for lymphatic/vascular invasions, and positive vertical margin in ESD specimens.

This study protocol was approved by the Institutional Review Board of Dokkyo Medical University, Tochigi, Japan, for clinical research (Registration No. R23-3J). This study was conducted in compliance with the revised Helsinki Declaration (1989). Regarding the patients’ informed consent, as this study was retrospective, the documents approved by the ethics committee were posted on the hospital website. We disclosed the information about this study and provided the patients with an opportunity to refuse. 

### 2.2. Methods

This retrospective cohort study extracted the following data from the patients’ electronic medical records and the endoscopy database: clinical findings (age, sex, body mass index, history of alcohol consumption, history of smoking), pre-ESD *H. pylori* infection status, endoscopic findings (location of initial neoplasm (U, upper part; M, middle part; L, lower part), presence of atrophic change [yes vs. no] and their scope), and histological findings (intestinal metaplasia, tumor size, histological type, invasion depth, R0 resection, synchronous multicentric cancers [yes vs. no]). Atrophy of gastric mucosa was determined by endoscopy was classified into three groups according to the Kimura and Takemoto classification as follows: no atrophy, closed type (mild), and open type (severe) [20].

This study included 369 patients with 382 SGEN lesions. Thirteen patients had two SGENs. A lesion with a larger diameter or deeper invasion was regarded as the initial SGENs for each of the thirteen patients.

We not only included patients with early gastric cancer in this study, but also included those with adenoma. According to Correa’s multi-stage cascade of gastric oncogenesis, prolonged mucosal inflammation causes changes in atrophy and intestinal metaplasia [21]. Further progression leads to low- or high-grade intraepithelial neoplasia. Gastric adenoma is classified as low- or high-grade intraepithelial neoplasia in the Revised Vienna classification [22]. Gastric adenoma is an advanced precancerous lesion that may progress into invasive gastric cancer [21]. Previous studies showed that gastric adenomas occasionally coexist with mucosal gastric cancer and demonstrated neoplastic progression to invasive carcinomas [23,24]. Additionally, a recent study showed that 34% of gastric adenomas progressed to gastric cancer lesions in the period of follow-up [25]. We reported diagnostic difficulty in differentiating gastric adenoma from early gastric cancer [26]. Endoscopic resection for superficial gastric neoplasms can be performed safely and gastric adenomas are usually a good candidate for endoscopic resection in Japan. As mentioned previously, gastric adenomas have background gastric mucosa which has a high risk of developing metachronous cancer as with early gastric cancer. Therefore, we included cases of gastric adenomas in this study.

Regular endoscopic surveillance was performed after ESD every 6 months to 1 year. According to a study on MSGEN, secondary SGENs identified within 1 year after endoscopic treatment were defined as synchronous because synchronous multiple tumors might have been overlooked during the initial ESD. Thus, SGENs identified > 1 year after endoscopic treatment were defined as MSGENs [27]. 

Spontaneous disappearance of *H. pylori* was defined as having no history of *H. pylori* therapy and testing negative for *H. pylori* infection but having severe mucosal atrophy corresponding to open-type atrophic gastritis (the Kimura–Takemoto classification) [13,20].

To elucidate the relationship between post-ESD MSGENs and *H. pylori* eradication therapy, we additionally established a matched not-MSGEN (Mnot-MSGEN) group, which was matched with the MSGEN group according to the clinicopathological risk factors identified in this study, age, and sex. Subsequently, we performed a statistical analysis based on whether *H. pylori* therapy was performed (yes vs. no) and the age at which *H. pylori* therapy was performed.

### 2.3. Confirmation of H. pylori Status

Serum antibody testing (Eiken Chemical, Tokyo, Japan) was performed for patients without *H. pylori* therapy before ESD. Moreover, the urea breath test (UBT; Otsuka Pharmaceutical, Tokyo, Japan) was performed for patients with *H. pylori* therapy before ESD to identify infection at the time. A positive result in at least one of these tests was considered evidence of *H. pylori* infection. Post-ESD *H. pylori* therapy was performed on patients who tested positive for *H. pylori* infection. The primary *H. pylori* therapy method was performed using combination therapies using a proton pump inhibitor (PPI; Lansoprazole 60 mg [Takeda Pharmaceutical, Tokyo, Japan]) or a potassium-competitive acid blocker (Vonoprazan 40 mg [Takeda Pharmaceutical]), and the two antibiotics Amoxicillin 1500 mg (Takeda Pharmaceutical) and Clarithromycin 400 mg (Takeda Pharmaceutical). The secondary *H. pylori* therapy method was applied in case the primary *H. pylori* therapy method had not been successful. The secondary *H. pylori* therapy method was performed as combination therapies using Lansoprazole 60 mg (Takeda Pharmaceutical) or Vonoprazan 40 mg (Takeda Pharmaceutical), and the two antibiotics, Amoxicillin 1500 mg (Takeda Pharmaceutical) and metronidazole 500 mg (Takeda Pharmaceutical). The success or failure of *H. pylori* therapy was evaluated after 4 to 6 months using UBT or serum antibody testing. We collected data regarding *H. pylori* status, including whether patients had a history of *H. pylori* therapy, from their medical records.

### 2.4. Histopathologic Analysis

We used the Japanese Classification of Gastric Carcinoma, 15th edition [28], to determine the histopathologic diagnosis of early gastric cancer, location (U; M, L, lower), cancerous histological type (Differentiated (tub1/tub2), Undifferentiated (por/sig), invasion depth (M, mucosal layer; SM, submucosal layer), ulcer (yes vs. no), and lymphatic/venous invasion. We defined early gastric cancer as the invasion being limited to the submucosa (SM). Gastric adenoma was classified into tub1 and mucosal cancer in this study. Histological intestinal metaplasia (yes vs. no) was determined using hematoxylin and eosin staining and periodic acid–Schiff staining of background mucosa in ESD specimens. One pathologist who has expertise in gastrointestinal tumors (H.Y.) diagnosed all pathologic findings.

### 2.5. Statistical Analyses

We performed statistical analysis designed to identify risk factors for the MSGEN group using the *t*-test/Mann–Whitney U test and the Chi-squared test for continuous and categorical variables, respectively. We calculated the cumulative incidence of MSGENs using the Kaplan–Meier method in order to confirm the risks. The follow-up period was calculated from the day of the initial ESD. Regular endoscopic surveillance (every 6 months to 1 year) was performed after ESD; moreover, the follow-up period ended upon MSGENs occurrence. The incidence of MSGENs was analyzed using the person–years method. Univariate analysis was performed using the log-rank test; moreover, we longitudinally analyzed the relation between identified significant factors with significant and MSGEN development. Statistical significance was set at *p*-value < 0.05. All statistical analyses were performed using SPSS version 20 (SPSS Japan Inc., Tokyo, Japan).

## 3. Results

Table 1 shows the clinical findings for the 369 patients included. The median age (IQR) was 73 (67–79) years, and the male-to-female ratio was 2.3:1. There were 369 SGENs lesions, 66 histological adenoma lesions, and 303 adenocarcinoma lesions (M: 264 lesions, SM: 39 lesions) that underwent ESD. We found twenty-seven patients (27 lesions; 7.0%: two patients with adenomas, twenty-five patients with carcinoma) with post-ESD MSGENs. Eleven or 2/14 lesions of MSGEN were located in U, M/L parts. Table 2 shows the clinicopathological findings for the 342 and 27 patients in the not-MSGEN and MSGENs groups, respectively.

Table 2 demonstrates a comparison of clinicopathologic factors between the “not-MSGEN” and “MSGEN” groups. There was a significantly higher incidence of MSGENs in cases of histological intestinal metaplasia (HIM; *p* = 0.04) and initial neo-plasm location in the U or M parts (INUM; *p* = 0.04). There was also a significant difference between the locations (U or M/L) of the initial SGENs and MSGENs (10 or 13/4 vs. 11 or 2/14: *p* < 0.01).

**Figure 2:**
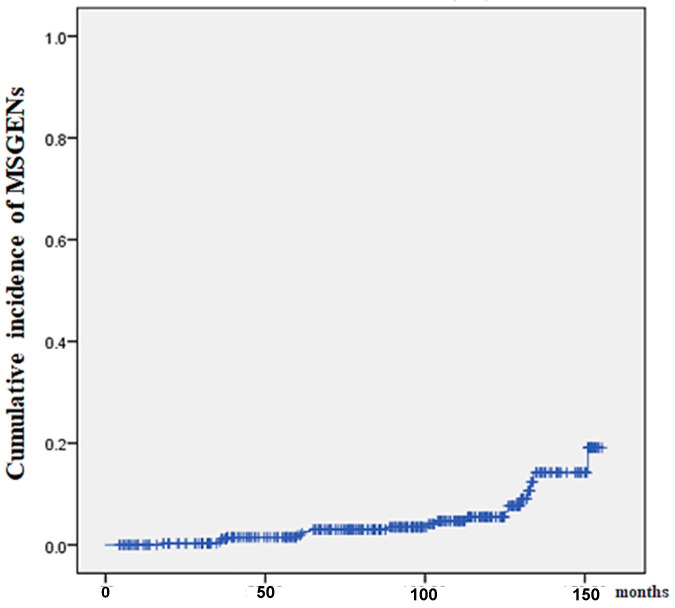
Kaplan–Meier analysis for the cumulative incidence of MSGEN after ESD.

Figure 2 shows the results of our analysis of the cumulative incidence of MSGENs using the Kaplan–Meier method. The median duration (IQR) from initial ESD to MSGENs was 95 (54–127) months. The shortest and longest durations from ESD to MSGENs detection were 18 months and 151 months, respectively.

**Figure 3:**
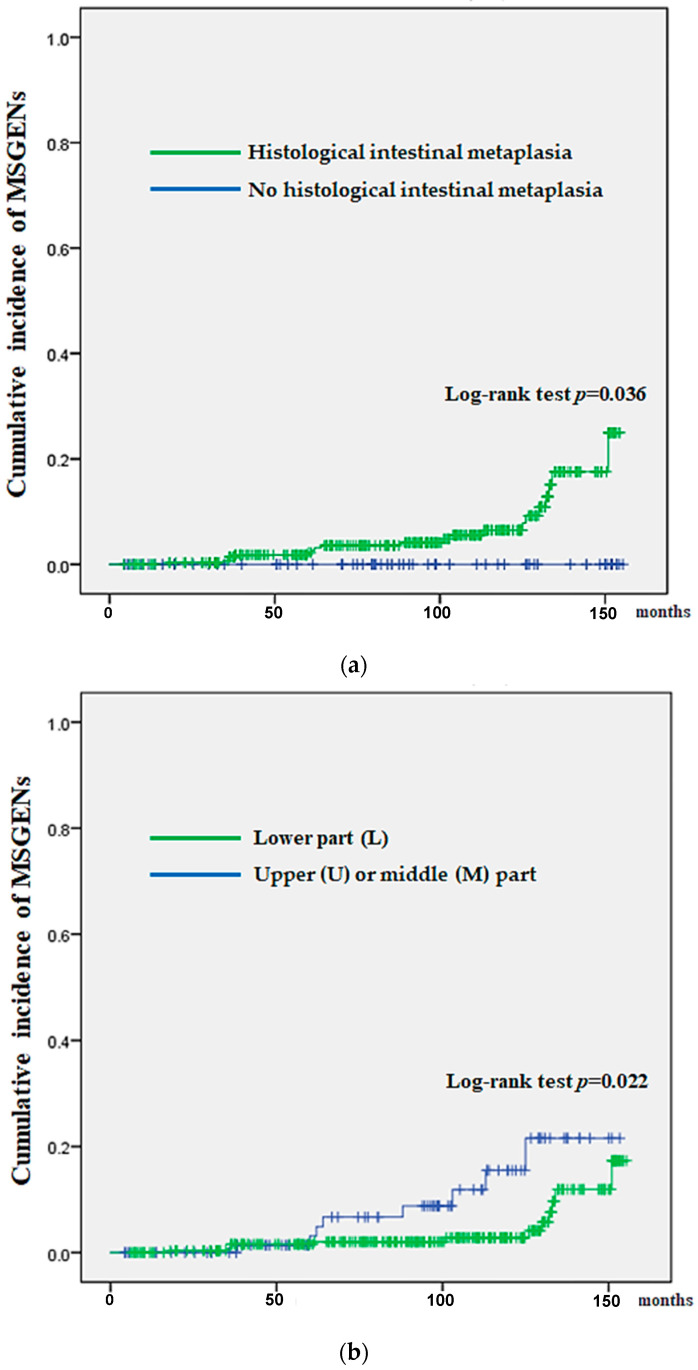
Kaplan–Meier analysis for the cumulative incidence of MSGEN by histological intestinal metaplasia (**a**), and location of initial SGEN (**b**). (**a**): Histological intestinal metaplasia (presence vs. absence). (**b**): Location of initial SEGN (U/M vs. L).

Comparisons after classifying the 369 patients based on the HIM (yes vs. no) and the initial neoplasm location (U/M vs. L) revealed a significantly higher cumulative incidence in the HIM (*p* = 0.036) and INUM groups (*p* = 0.022) (Figure 3a and Figure 3b, respectively).

We extracted 27 patients from the not-MSGEN group and matched them to the MSGENs group according to HIM, INUM, age, sex, and histological type (i.e., Mnot-MSGEN group). Table 3 shows a comparison of the clinicopathologic factors between the Mnot-MSGENs and MSGENs groups. No significant between-group difference was found for variables unrelated to *H. pylori* therapy. Regarding *H. pylori* therapy, there was no significant between-group difference in the age at the time of *H. pylori* therapy (*p* = 0.261). All *H. pylori*-positive patients received successful *H. pylori* therapy. Compared with the Mnot-MSGENs group, the MSGENs group had a significantly higher number of patients with spontaneous disappearances of *H. pylori* (i.e., patients who have no history of *H. pylori* therapy and negative tests for *H. pylori* infection but severe atrophy of their gastric mucosa) (*p* = 0.003).

**Figure 4:**
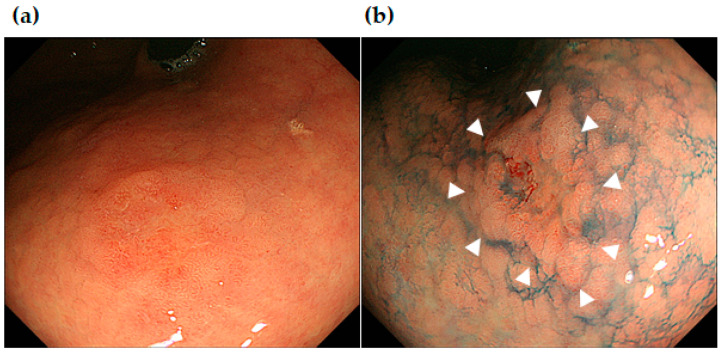
Initially developed early gastric cancer (tub 1, white arrows in (**b**). Endoscopic images of white light endoscopy (**a**) and indigocarmine chromoendoscopy (**b**) which were taken prior to the initial ESD.

**Figure 5:**
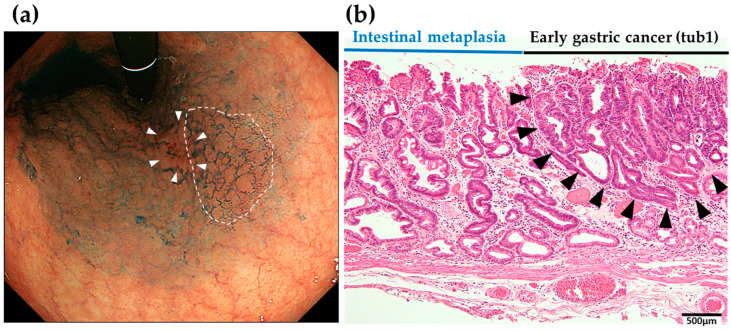
(**a**) Endoscopic images of intestinal metaplasia (granular surface within the white dashed line) found around the initially developed early gastric cancer (white arrows). (**b**) Histological findings at the boundary of the peritumor region of intestinal metaplasia and the early gastric cancer (the extent of well-differentiated tubular adenocarcinoma in the mucosal layer indicated by black arrows).

**Figure 6:**
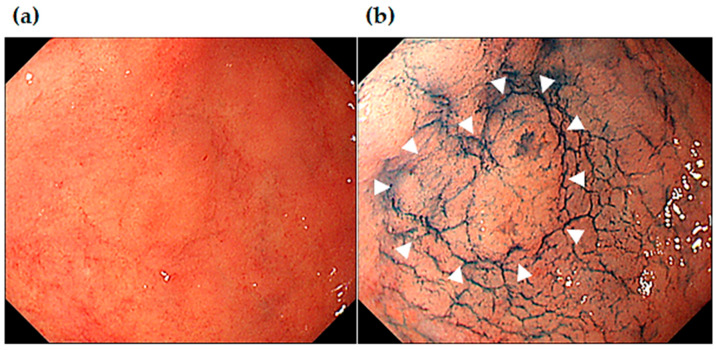
Endoscopic images of MSGEN, early gastric cancer (tub 1), which developed in the same patient as seen in Figure 4 and Figure 5. Endoscopic images of white light endoscopy (**a**) and indigocarmine chromoendoscopy ((**b**): white arrows indicating the early cancer lesion) taken prior to the second ESD.

Figure 4, Figure 5 and Figure 6 show some representative cases of post-ESD MSGENs. There is a depressed lesion (0-IIc) measuring 12 mm in diameter and presenting a reddish coloration associated with a marginal elevation in the lesser curvature of the upper (U) part (Figure 4a,b). In the region surrounding the depressed lesion, there are granular mucosal changes (indicated by the dashed line in Figure 5a) and atrophic changes over a wide area that is associated with a visible vascular pattern, which are consistent with the endoscopic appearance of intestinal metaplasia (Figure 5a). Histological findings identified in the ESD specimens indicated differentiated adenocarcinoma (tub1) localized within the mucosa; moreover, both vascular invasion and the resection stump were negative, which indicated complete curative resection (Figure 5b).

Figure 6 shows endoscopic images of an MSGEN which developed 37 months after ESD in the same patient that can be seen in Figure 4 and Figure 5. The endoscopic images show a slightly elevated (0-IIa) 7 mm lesion in the angle of the lesser curvature of the stomach. It was difficult to identify in the observation under white light endoscopy since it had unclear borders. Indigocarmine chromoendoscopy made the borders clear, which allowed for it to be identified as a slightly elevated lesion. The histology of the ESD resection sample showed differentiated adenocarcinoma (tub1) confined to the mucosal layer; moreover, it was negative for both vascular invasion and resection margins, which indicated complete curative resection (R0 resection).

## 4. Discussion

Our findings suggest that HIM and INUM are independent clinicopathological risk factors for MSGENs in patients who underwent ESD for SGENs. This is because the patients with the factors have a significantly higher cumulative incidence of MSGENs compared to those without the factors. After group-matching according to these two risk factors, our analysis revealed a significant association between development of MSGENs and the spontaneous disappearance of *H. pylori*. 

Our identified risk factors, HIM and INUM, will be influenced by long-term *H. pylori* infection [29] because HIM could be caused by decreased gastric motility and increased intestinal fluid reflux due to long-term *H. pylori* infection [30]. Although we utilized histological confirmation, intestinal metaplasia can be diagnosed with a high accuracy by using magnification endoscopy or image enhanced endoscopy such as linked color imaging [31,32]. This suggests that the degree of risk for developing MSGENs can be estimated by endoscopic inspection. 

Regarding the other risk factor, INUM, there is a strong association with atrophic mucosa, a precancerous condition, which may develop over a wide area from the middle (M) to the upper (U) part surrounding the initial SGEN lesion. A longer period of *H. pylori* infection is related to the greater expansion of mucosal atrophy. Consequently, a greater expansion of atrophic mucosa can bring a higher risk of cancer development [33]. This suggests that a greater expansion of atrophic mucosa may be associated with the higher risk of MSGENs onset after ESD. 

Studies have shown that severe atrophic changes (i.e., wide expansion of atrophic mucosa) could also increase the risk of initial gastric cancer onset [34,35,36,37,38]. This could be attributed to the chronic inflammation related to *H. pylori* infection causing the accumulation of various DNA methylation abnormalities, with the degree being correlated to the risk of gastric cancer onset [39,40]. Additionally, the mucosa remaining after ESD in patients with gastric cancer who have accumulated DNA methylation abnormalities caused by chronic inflammation could be at high risk of cancer onset. Our finding on HIM/INUM and previous studies’ results suggest that severe atrophic changes may be a risk factor for the initial onset of both SGENs and MSGENs. 

International research showed that the spontaneous disappearance of *H. pylori* is associated with a high risk of initial gastric cancer onset [13,40]. This study showed that the spontaneous disappearance of *H. pylori* was significantly associated with the development of MSGENs after ESD. In this study, all nine of the patients with spontaneous disappearance of *H. pylori* had the most severe open-type (grade O3) atrophic gastritis. Although the grades of gastric mucosal atrophy (no/closed/open) were not significantly different according to the frequencies of the development of MSGENs, the most severe atrophic gastritis (grade O3) in the spontaneous disappearance cases may generate a strong effect on the significant differences in the MSGENs (4% vs. 33%). This is because the most severe atrophic gastritis (grade O3) results in very long-term continuous chronic inflammation, which results in highly advanced DNA methylation abnormalities that could contribute to the onset of MSGENs in this study. 

Considering the risk factors identified in this study and the accumulation of DNA methylation, *H. pylori* eradication therapy could contribute to reducing the risk of developing initial gastric cancer [33] as well as reducing the risk of MSGENs onset [4] following ESD. Although *H. pylori* therapy has been widely used across the world, a recent systematic review demonstrated that there was no significant decrease in the overall infection rates of *H. pylori* in the periods between 2000–2008 and 2009–2016, with the rates being 46.8% and 44.9%, respectively [41]. Therefore, *H. pylori* eradication therapy should be performed in SGEN patients who have been proven to be positive for *H. pylori* infection as soon as possible. Earlier eradication in *H. pylori*-positive patients before the development of atrophic gastritis may facilitate the prevention of post-ESD MSGENs.

According to this study, the median period for the onset of multiple tumors was 95 months; moreover, the longest amount of time was 151 months, which is over 12 years. Since 18 patients underwent ESD for MSGENs during the observation period in this study, MSGENs onset can occur even >12 years after ESD. This suggests that we should perform surveillance endoscopies after ESD over long periods of time 10 years. 

A large-scale study showed that more than 95% of metachronous multiple gastric cancers can be treated with endoscopic resection by follow-up through annual endoscopic surveillance [42]. In this study, regular endoscopic surveillance was performed after ESD every six months or once a year. Twenty-seven MSGENs were detected as being adenoma (*n* = 2) or intramucosal adenocarcinomas of differentiated type (*n* = 25), and we achieved curative resections for all of the MSGENs by ESD. Thus, endoscopic surveillance at least once a year will be acceptable for patients who received ESD for SGENs. Particularly for the patients with significant risk factors suggested in this study, endoscopic surveillance every six months may be favorable.

This study has several limitations. First, this was a single-center retrospective study with a relatively small number of patients with MSGENs. Moreover, the number of MSGEN cases was too small to perform matched analysis. The small number of cases may affect the statistical power of the analysis and the validity of the results. Therefore, this study’s results need to be confirmed by a further large-scale multicenter study. Second, it was difficult to survey the time frame for the establishment of the *H. pylori* infection even though this could have been achieved by conducting a follow-up survey; therefore, it was impossible to accurately assess the period of infection. Third, we did not have a biopsy protocol for assessing the extent of atrophic gastritis/HIM in the non-neoplastic gastric mucosa and a pepsinogen test was only performed for some of the patients to estimate the degree of gastric mucosal atrophy. Fourth, we did not measure the accumulation of DNA methylation abnormalities. Fifth, we did not evaluate other associations with the development of MSGENs such as genetic predispositions (e.g., CDH-1 gene mutation in hereditary diffuse gastric cancer and APC exon 1B mutations in gastric adenocarcinoma and proximal polyposis) and dietary factors (nitrate or nitrite, pickled vegetables, and salted fish), and comorbidities (e.g., asbestos and EBV infection) [43]. Sixth, the identified risk factors for HIM, INUM, and the spontaneous disappearance of *H. pylori* can be putative confounders for each other because all of them are closely associated with severe atrophic gastritis and long-term *H. pylori* infection. Finally, regarding the secondary endpoint, we only performed a χ^2^ test in the comparison of cumulative incidences of MSGEN between the two patient groups (with/without spontaneous disappearance of *H. pylori*). This is because two Kaplan–Meier curves of cumulative incidences of MSGEN in the two patient groups (with/without spontaneous disappearance of *H. pylori*) crossed. When two Kaplan–Meier curves cross each other, it may have be difficult to use the log-rank test and hazard ratio to properly assess the difference between the two groups. Although the studies proposed special tests for the crossed Kaplan–Meier curves, the evidence level will now be low [44,45]. The aforementioned small number of patients who experienced the spontaneous disappearance of *H. pylori* (*n* = 10) may affect that the two Kaplan–Meier curves crossed and unavailability to use the statistical analysis.

In conclusion, this study suggested that HIM and INUM were independent clinicopathological risk factors for developing MSGENs after ESD for SGENs. Additionally, the spontaneous disappearance of *H. pylori* may be a risk factor for MSGENs in the ESD patients who have HIM and INUM. A larger prospective trial with long-term follow-up is warranted to validate the three risk factors in clinical practice to overcome the limitations of this study. 

## Figures and Tables

**Table 1:** Characteristics of patients and lesions.

Sex, Male: Female; *n* (%)	257 (70):112 (30)
Age, median (IQR)	73 (67–79)
Adenoma: adenocarcinoma, *n* (%)	66 (18): 303 (82)
Size of initial SGENs: mm, median (IQR)	15 (10–23)
Location of initial SGENs U:M:L, *n* (%)	88 (24):133 (36):148 (40)
MSGENs, *n* (%)	27 (7.1)
Location of MSGENs U:M:L, *n* (%)	11 (41):2 (7):14 (52)

IQR, interquartile range; SGENs, superficial gastric epithelial neoplasms; U/M/L; the upper/middle/lower area; MSGENs, metachronous superficial gastric epithelial neoplasms.

**Table 2:** Comparison of clinicopathologic factors between the “not-MSGEN” and “MSGEN” group.

Findings	Not-MSGEN *n* = 342	MSGEN *n* = 27	*p*
Age, years; median (IQR)	73 (66–78)	77 (72–81)	0.20 ^#^
Sex, male/female; *n*	235/107	22/5	0.32 ^†^
Body mass index, median (IQR)	24 (21–26)	24 (20–26)	0.35 ^‡^
Alcohol drinking, *n* (%)	141 (41)	12 (44)	0.84 ^†^
Smoking, *n* (%)	181 (53)	13 (48)	0.29 ^†^
Gastric mucosal atrophy (no/closed/open)	10/29/303	0/0/27	0.24 ^†^
Histological intestinal metaplasia (HIM), *n* (%)	279 (82)	27 (100)	0.04 ^†^
*Hp* infection, current/past or negative, *n* (%)	213 (62)/102 (30) or 27 (8)	16 (59)/2 (7) or 9 (34)	0.05 ^†^
Location of initial SGENs (U, M/L)	78, 120/144	10, 13/4	0.04 ^†^
Histologic type (differentiated/undifferentiated) *	330/12	27/0	0.08 ^†^
Size, mm; median (IQR)	15 (10–23)	12 (10–19)	0.08 ^#^
Depth of initial tumor, M/SM	305/37	25/2	0.44 ^†^
Ulcer (−/+)	310/32	23/4	0.50 ^†^
Lymphovascular invasion (−/+)	342/0	27/0	0.78 ^†^
Vessel invasion (−/+)	342/0	27/0	0.97 ^†^

IQR, interquartile range; *Hp*, *Helicobacter pylori*; SGENs, superficial gastric epithelial neoplasms; U/M/L, the upper/middle/lower parts; M/SM, mucosal/submucosal layers. * Adenoma classified into differentiated cancer (tub1) and tumor depth of M; ^†^ χ^2^ test; ^‡^ *t*-test; ^#^ Mann–Whitney U-test.

**Table 3:** Comparison of clinicopathologic factors between adjusted “Mnot-MSGEN” and “MSGEN” groups.

Findings	Mnot-MSGEN *n* = 27	MSGEN *n* = 27	*p*
Age, years; median (IQR)	70 (66–78)	77 (72–81)	0.29 ^#^
Sex, male/female; *n*	23/4	22/5	0.72 ^†^
Body mass index, median (IQR)	23 (17–30)	24 (20–226)	0.76 ^‡^
Alcohol drinking, *n* (%)	10 (37)	12 (44)	0.31 ^†^
Smoking, *n* (%)	16 (59)	13 (48)	0.26 ^†^
Gastric mucosal atrophy (no/closed/open)	3/1/23	0/0/27	0.06 ^†^
Histological intestinal metaplasia (HIM), *n* (%)	21 (78)	27 (100)	0.09 ^†^
*Hp* infection, current/past or negative, *n* (%)	19 (70)/7 (26) or 1 (4)	16 (59)/2 (7) or 9 (34)	0.45 ^†^
Location of initial SGENs (U, M/L)	8, 10/9	10, 13/4	0.21 ^†^
Histologic type (differentiated/undifferentiated) *	25/2	27/0	0.49 ^†^
Size, mm; median (IQR)	15 (10–23)	12 (10–19)	0.57 ^#^
Depth of initial tumor, M/SM	26/1	25/2	0.09 ^†^
Ulcer (−/+)	24/3	23/4	0.69 ^†^
Lyphovascular invasion (−/+)	27/0	27/0	-
Vessel invasion (−/+)	27/0	27/0	-
Synchronous SGEN, *n* (%)	0 (0.0)	2 (7.4)	0.07 ^†^
Age of *Hp* therapy, years (IQR)	73 (63–82)	70 (63–77)	0.261 ^‡^
Spontaneous disappearance of *Hp*, *n* (%) **	1 (4)	9 (33)	0.003 ^†^

IQR, interquartile range; *Hp*, *Helicobacter pylori*; SGENs, superficial gastric epithelial neoplasms; U/M/L, the upper/middle/lower parts; M/SM, mucosal/submucosal layers. * Adenoma classified into differentiated cancer (tub1) and tumor depth of M; ** *Hp*-negative patients with no history of *Hp* therapy but having severe atrophy; ^†^ χ^2^ test; ^‡^ *t*-test; ^#^ Mann–Whitney U-test.

## Data Availability

Data is unavailable due to privacy or ethical restrictions.

## References

[1] Freddie B., Jacques F., Isabelle S., Rebbeca L., Lindsey A., Ahmedin J. (2018). Global cancer statistics 2018: GLOBOCAN estimates of incidence and mortality worldwide for 36 cancers in 185 countries. Cancer J. Clin..

[2] Ono H., Yao K., Fujishiro M., Oda I., Uedo N., Nimura S., Yahagi N., Iishi H., Oka M., Ajioka Y. (2021). Guidelines for endoscopic submucosal dissection and endoscopic mucosal resection for early gastric cancer (second edition). Dig. Endosc..

[3] Shiotani A., Haruma K., Graham D.Y. (2014). Metachronous gastric cancer after successful *Helicobacter pylori* eradication. World J. Gastroenterol..

[4] Fukase K., Kato M., Kikuchi S., Inoue K., Uemura N., Okamoto S., Terao S., Amagai K., Hayashi S., Asaka M. (2008). Effect of eradication of *Helicobacter pylori* on incidence of metachronous gastric carcinoma after endoscopic resection of early gastric cancer: An open-label, randomized controlled trial. Lancet.

[5] Maehata Y., Nakamura S., Fujisawa K., Esaki M., Moriyama T., Asano K., Fuyuno Y., Yamaguchi K., Egashira I., Kim H. (2012). Long-term effect of *Helicobacter pylori* eradication on the development of metachronous gastric cancer after endoscopic resection of early gastric cancer. Gastrointest. Endosc..

[6] Choi J., Kim S.G., Yoon H., Im J.P., Kim J.S., Kim W.H., Jung H.C. (2014). Eradication of *Helicobacter pylori* after endoscopic resection of gastric tumors does not reduce incidence of metachronous gastric carcinoma. Clin. Gastroenterol. Hepatol..

[7] Choi I.J., Kook M.C., Kim Y.I., Cho S.J., Lee J.Y., Kim C.G., Park B., Nam B.H. (2018). *Helicobacter pylori* therapy for the prevention of metachronous gastric cancer. N. Engl. J. Med..

[8] Yoo H.W., Hong S.J., Kim S.H. (2023). *Helicobacter pylori* treatment and gastric cancer risk after en-doscopic resection of dysplasia: A nationwide cohort study. Gastroenterology.

[9] Noh C.K., Lee E., Park B., Lim S.G., Shin S.J., Lee K.M., Lee G.H. (2023). Effect of *Helicobacter pylori* eradication treatment on meta-chronous gastric neoplasm prevention following endoscopic submucosal dissection for gastric adenoma. J. Clin. Med..

[10] Kato M., Nishida T., Yamamoto K., Hayashi S., Kitamura S., Yabuta T., Yoshio T., Nakamura T., Komori M., Kawai N. (2013). Scheduled endoscopic surveillance controls secondary cancer after curative endoscopic resection for early gastric cancer: A multicenter retrospective cohort study by Osaka University ESD study group. Gut.

[11] Ono S., Kato M., Suzuki M., Ishigaki S., Takahashi M., Haneda M., Mabe K., Shimizu Y. (2012). Frequency of *Helicobacter pylori*-negative gastric cancer and gastric mucosal atrophy in a Japanese endoscopic submucosal dissection series including histological, endoscopic and serological atrophy. Digestion.

[12] Boda T., Ito M., Yoshihara M., Kitamura Y., Matsuo T., Oka S., Tanaka S., Chayama K. (2013). Advanced method for evaluating of gastric cancer risk by serum marker: Determination of true low-risk subjects for gastric neoplasia. Helicobacter.

[13] Kishikawa H., Ojiro K., Nakamura K., Katayama T., Arahata K., Takarabe S., Miura S., Kanai T., Nishida J. (2020). Previous *Helicobacter pylori* infection–induced atrophic gastritis: A distinct disease entity in an understudied population without a history of eradication. Helicobacter.

[14] Ikeda F., Shikata K., Hata J., Fukuhara M., Hirakawa Y., Ohara T., Mukai N., Nagata M., Yoshida D., Yonemoto K. (2016). Combination of *Helicobacter pylori* antibody and serum pepsinogen as a good predictive tool of gastric cancer incidence: 20-year prospective data from the *Hisayama* study. J. Epidemiol..

[15] Watabe H., Mitsushima T., Yamaji Y., Okamoto M., Wada R., Kokubo T., Doi H., Yoshida H., Kawabe T., Omata M. (2005). Predicting the development of gastric cancer from combining *Helicobacter pylori* antibodies and serum pepsinogen status: A prospective endoscopic cohort study. Gut.

[16] Yamaji Y., Watabe H., Yoshida H., Kawabe T., Wada R., Mitsushima T., Omata M. (2009). High-risk population for gastric cancer development based on serum pepsinogen status and lifestyle factors. Helicobacter.

[17] Ohata H., Kitauchi S., Yoshimura N., Mugitani K., Iwane M., Nakamura H., Yoshikawa A., Yanaoka K., Arii K., Tamai H. (2004). Progression of chronic atrophic gastritis associated with *Helicobacter pylori* infection increases risk of gastric cancer. Int. J. Cancer.

[18] Kaji K., Hashiba A., Uotani C., Yamaguchi Y., Ueno T., Ohno K., Takabatake I., Wakabayashi T., Doyama H., Ninomiya I. (2019). Grading of atrophic gastritis is useful for risk stratification in endoscopic screening for gastric cancer. Am. J. Gastroenterol..

[19] Take S., Mizuno M., Ishiki K., Yoshida T., Ohara N., Yokota K., Oguma K., Okada H., Yamamoto K. (2011). The long-term risk of gastric cancer after the successful eradication of *Helicobacter pylori*. J. Gastrotenterol..

[20] Kimura K., Takemoto T. (1969). An endoscopic recognition of the atrophic border and its significance in chronic gastritis. Endoscopy.

[21] Correa P. (1992). Human gastric carcinogenesis: A multistep and multifactorial process—First American Cancer Society award lecture on cancer epidemiology and prevention. Cancer Res..

[22] Dixon M.F. (2002). Gastrointestinal epithelial neoplasia: Vienna revisited. Gut.

[23] Kamiya T., Morishita T., Asakura H., Miura S., Munakata Y., Tsuchiya M. (1982). Long-term follow-up study on gastric adenoma and its relation to gastric protruded carcinoma. Cancer.

[24] Rugge M., Cassaro M., Di Mario F., Leo G., Leandro G., Russo V.M., Pennelli G., Farinati F. (2003). The long term outcome of gastric non-invasive neoplasia. Gut.

[25] Okamoto Y., Kanzaki H., Tanaka T., Sakae H., Abe M., Iwamuro M., Kawano S., Kawahara Y., Okada H. (2021). Gastric Adenoma: A High Incidence Rate of Developing Carcinoma and Risk of Metachronous Gastric Cancer according to Long-Term Follow-Up. Digestion.

[26] Tamai N., Kaise M., Nakayoshi T., Katoh M., Sumiyama K., Gohda K., Yamasaki T., Arakawa H., Tajiri H. (2006). Clinical and endoscopic characterization of depressed gastric adenoma. Endoscopy.

[27] Mori G., Nakajima T., Asada K., Shimazu T., Yamamichi N., Maekita T., Yokoi C., Fujishiro M., Gotoda T., Ichinose M. (2016). Incidence of and risk factors for metachronous gastric cancer after endoscopic resection and successful *Helicobacter pylori* eradication: Results of a large-scale, multicenter cohort study in Japan. Gastric Cancer.

[28] Japanese Gastric Cancer Association (2017). Japanese Classification of Gastric Carcinoma.

[29] Kodama M., Murakami K., Okimoto T., Abe H., Sato R., Ogawa R., Mizukami K., Shiota S., Nakagawa Y., Soma W. (2013). Histological characteristics of gastric mucosa prior to *Helicobacter pylori* eradication may predict gastric cancer. Scand. J. Gastroenterol..

[30] Lahner E., Carabotti M., Annibale B. (2018). Treatment of *Helicobacter pylori* infection in atrophic gastritis. World J. Gastroenterol..

[31] Uedo N., Ishihara R., Iishi H., Yamada T., Imanaka K., Takeuchi Y., Higashino K., Ishiguro S., Tatsuta M. (2006). A new method of diagnosing gastric intestinal metaplasia: Narrowband imaging with magnifying endoscopy. Endoscopy.

[32] Ono S., Kato M., Tsuda M., Miyamoto S., Abiko S., Shimizu Y., Sakamoto N. (2018). Lavender Color in Linked Color Imaging Enables Noninvasive Detection of Gastric Intestinal Metaplasia. Digestion.

[33] Uemura N., Okamoto S., Yamamoto S., Matsumura N., Yamaguchi S., Yamakido M., Taniyama K., Sasaki N., Schlemper R.J. (2001). *Helicobacter pylori* infection and the development of gastric cancer. N. Engl. J. Med..

[34] Correa P. (1995). *Helicobacter pylori* and gastric carcinogenesis. Am. J. Surg. Pathol..

[35] Take S., Mizuno M., Ishiki K., Nagahara Y., Yoshida T., Yokota K., Oguma K. (2007). Baseline gastric mucosal atrophy is a risk factor associated with the development of gastric cancer after *Helicobacter pylori* eradication therapy in patients with peptic ulcer diseases. J. Gastroenterol. Hepatol..

[36] Sakitani K., Hirata Y., Watabe H., Yamada A., Sugimoto T., Yamaji Y., Yoshida H., Maeda S., Omata M., Koike K. (2011). Gastric cancer risk according to the distribution of intestinal metaplasia and neutrophil infiltration. J. Gastroenterol. Hepatol..

[37] Parisa K., Farhad I., Sharmila A., Neal D.F., Frain K. (2014). Gastric cancer: Descriptive epidemiology, risk factors, screening, and prevention. Cancer Epidemiol. Biomark. Prev..

[38] Asada K., Nakajima T., Shimazu T., Yamamichi N., Maekita T., Yokoi C., Oda I., Ando T., Yoshida T., Nanjo S. (2015). Demonstration of the usefulness of epigenetic cancer risk prediction by a multicentre prospective cohort study. Gut.

[39] Yamaguchi Y., Nagata Y., Hiratsuka R., Kawase Y., Tominaga T., Takeuchi S., Sakagami S., Ishida S. (2016). Gastric cancer screening by combined assay for serum anti-*Helicobacter pylori* IgG antibody and serum pepsinogen levels—The ABC method. Digestion.

[40] IARC Helicobacter pylori Working Group (2014). Helicobacter pylori Eradication as a Strategy for Preventing Gastric Cancer.

[41] Zamani M., Ebrahimtabar F., Zamani V., Miller W.H., Alizadeh-Navaei R., Shokri-Shirvani J., Derakhshan M.H. (2018). Systematic review with meta-analysis: The worldwide prevalence of *Helicobacter pylori* infection. Aliment. Pharmacol. Ther..

[42] Nakajima T., Oda I., Gotoda T., Hamanaka H., Eguchi T., Yokoi C., Saito D. (2006). Metachronous gastric cancers after endoscopic resection: How effective is annual endoscopic surveillance?. Gastric Cancer.

[43] Carneiro F., Fukayama M., Grabsch H.I., Yasui W. (2019). Gastric adenocarcinoma. WHO Classification of Digestive System Tumors.

[44] Dormuth I., Liu T., Xu J., Yu M., Pauly M., Ditzhaus M. (2022). Which test for crossing survival curves? A user’s guideline. BMC Med. Res. Methodol..

[45] Zheng S., Wang D., Qiu J., Chen T., Gamalo M. (2023). A win ratio approach for comparing crossing survival curves in clinical trials. J. Biopharm. Stat..

